# Ultrasound in juvenile idiopathic arthritis

**DOI:** 10.1186/s12969-016-0096-2

**Published:** 2016-05-27

**Authors:** Silvia Magni-Manzoni

**Affiliations:** Pediatric Rheumatology Unit, IRCCS Ospedale Pediatrico Bambino Gesù, Piazza Sant’Onofrio 4, 00165 Rome, Italy

**Keywords:** Musculoskeletal ultrasound, Juvenile idiopathic arthritis, Children, Pediatric rheumatology

## Abstract

**Background:**

In the recent years, musculoskeletal ultrasound (MSUS) has been regarded as especially promising in the assessment of juvenile idiopathic arthritis (JIA), as a reliable method to precisely document and monitor the synovial inflammation process.

**Main content:**

MSUS is particularly suited for examination of joints in children due to several advantages over other imaging modalities. Some challenges should be considered for correct interpretation of MSUS findings in children, due to the peculiar features of the growing skeleton. MSUS in JIA is considered particularly useful for its ability to detect subclinical synovitis, to improve the classification of patients in JIA subtypes, for the definition of remission, as guidance to intraarticular corticosteroid injections and for capturing early articular damage. Current evidence and applications of MSUS in JIA are documented by several authors. Recent advances and insights into further investigations on MSUS in healthy children and in JIA patients are presented and discussed in the present review.

**Conclusions:**

MSUS shows great promise in the assessment and management of children with JIA. Nonetheless, anatomical knowledge of sonographic changes over time, underlying immunopathophysiology, standardization and validation of MSUS in healthy children and in patients with JIA are still under investigation. Further research and educational efforts are required for expanding this imaging modality to more clinicians in their daily practice.

## Background

Juvenile idiopathic arthritis (JIA) is the most common chronic rheumatic disease of childhood and an important cause of acquired disability in children [1]. Despite the heterogeneity, all forms of JIA are characterized by prolonged synovial inflammation that can cause cartilage and bone damage, with severe impairment of physical function and impact on the quality of life. In the recent years, the availability of powerful and expensive drugs increased the need to identify patients with a high likelihood of developing erosive damage early and patients with a less aggressive disease, so as to institute the appropriate therapy at and for the most convenient time. This induced to search for sensitive methods for reliable documentation and precise monitoring of the synovial inflammation process [2–4]. Musculoskeletal ultrasound (MSUS) demonstrated to be a valid and reliable tool in the assessment of chronic inflammatory arthropathies in adults [5–7]. Therefore, it has been regarded as especially promising in the assessment of joints in children with JIA [8–11].

### Advantages of MSUS in children

**Fig. 1:**
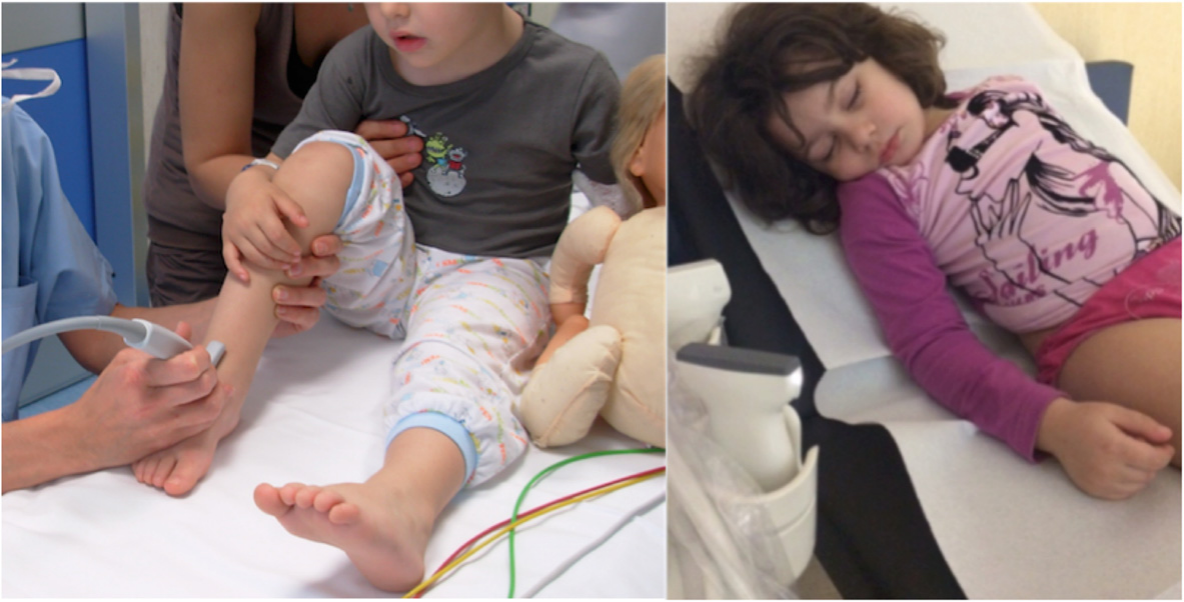
MSUS is well accepted by children and their parents, and can be performed as completion of the joint examination during the standard clinical assessment of the child. Some children have fun during MSUS evaluation, others totally relax and can fall asleep

Though MSUS has some limitations, it is particularly suited for use in children for several advantages over other imaging techniques (Table 1). It is quick, it does not expose the child to ionizing radiation, it does not require sedation or general anesthesia, it allows for multisite assessment in the same session, comparison between symptomatic and asymptomatic sites, dynamic study, and it is easily repeatable. Moreover, it is well accepted by both children and their parents, and it is the only imaging technique that can be coupled with the conventional approach to patient assessment in the clinic (Fig. 1).

**Table 1:** Advantages and limitations of musculoskeletal ultrasound (MSUS) compared to magnetic resonance imaging (MRI) and conventional radiology in children with juvenile idiopathic arthritis

Imaging modality	Advantages	Limitations
MSUS	Lack of exposure to ionizing radiation Rapidity of performance Ease of repeatability High patient acceptability Demonstration of soft tissue inflammation Direct visualization of cartilage Early detection of bone erosions Ability to scan multiple joints in a single session Support in guidance of procedures (e.g. intra- articular corticosteroid injections) Relatively inexpensive	Difficulties in carrying out in case of severe joint limitation Relatively small field of view Inability to assess the whole joint space Acoustic shadowing from overlying bones Limited value in the assessment of axial skeleton and temporomadibular joints Dependency on the properties and sensitivity of the ultrasound equipment Need of continuous practice after appropriate training Reliability, standardization and validation in children under investigation
MRI	Lack of exposure to ionizing radiation Multiplanar tomographical imaging Ability to assess the whole joint space Demonstration of soft tissue inflammation Direct visualization of cartilage Early detection of bone erosions Visualization of bone marrow oedema High tissue contrast Suitable for assessment of axial skeleton and temporomadibular joints	Intravenous contrast agent often required Possible allergic reaction to contrast agents General anesthesia required in younger children Long examination time Evaluation limited to one target joint Reliability, standardization and validation in children under investigation High cost Variable availability worldwide
Conventional radiology	Rapidity of performance Applicability to all joints Demonstration of joint space narrowing, disturbances of bone growth and maturation Detection of bone erosions Validated scoring methods in children Suitable for longitudinal evaluation of damage progression Low cost Widespread availability	Exposure to ionizing radiations Inability to directly visualize cartilage and soft tissue inflammation Late detection of bone erosions and joint space narrowing Projectional superimposition

### Challenges with MSUS in children

**Fig. 2:**
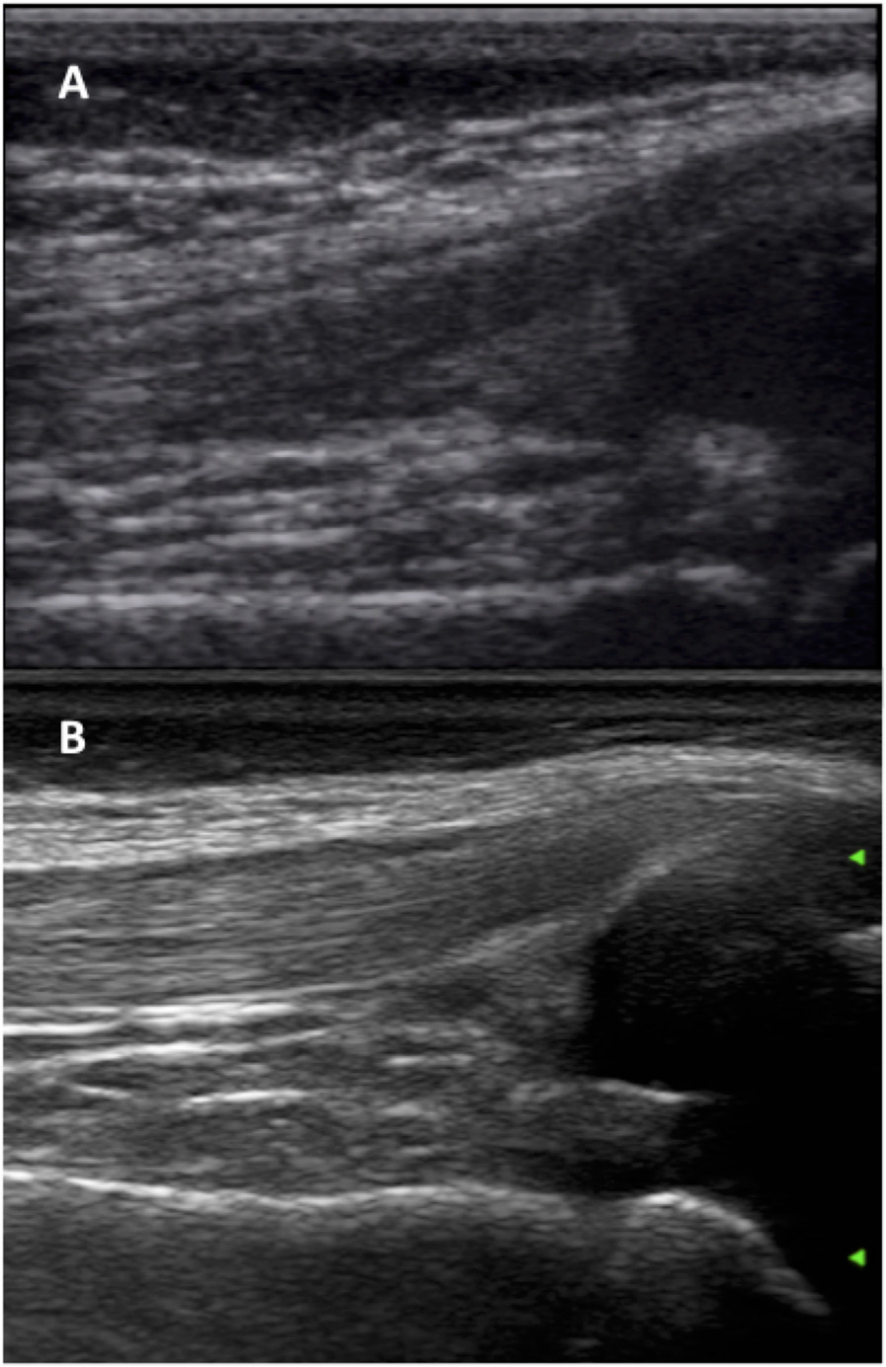
Longitudinal scan of the suprapatellar recess in two 7 years old boys performed with high multifrequency probe: **a**. Top-level ultrasound machine in the early 2000’s; **b**. Top-level ultrasound machine in 2015

Like other imaging modalities, MSUS is an operator dependent imaging technique. Further, it is highly dependent on the properties and sensitivity of the machine used, which can range widely (Fig. 2). As in adults, MSUS in children requires continuous practice after appropriate training and has a limited value in some musculoskeletal areas, such as the axial skeleton.

**Fig. 3:**
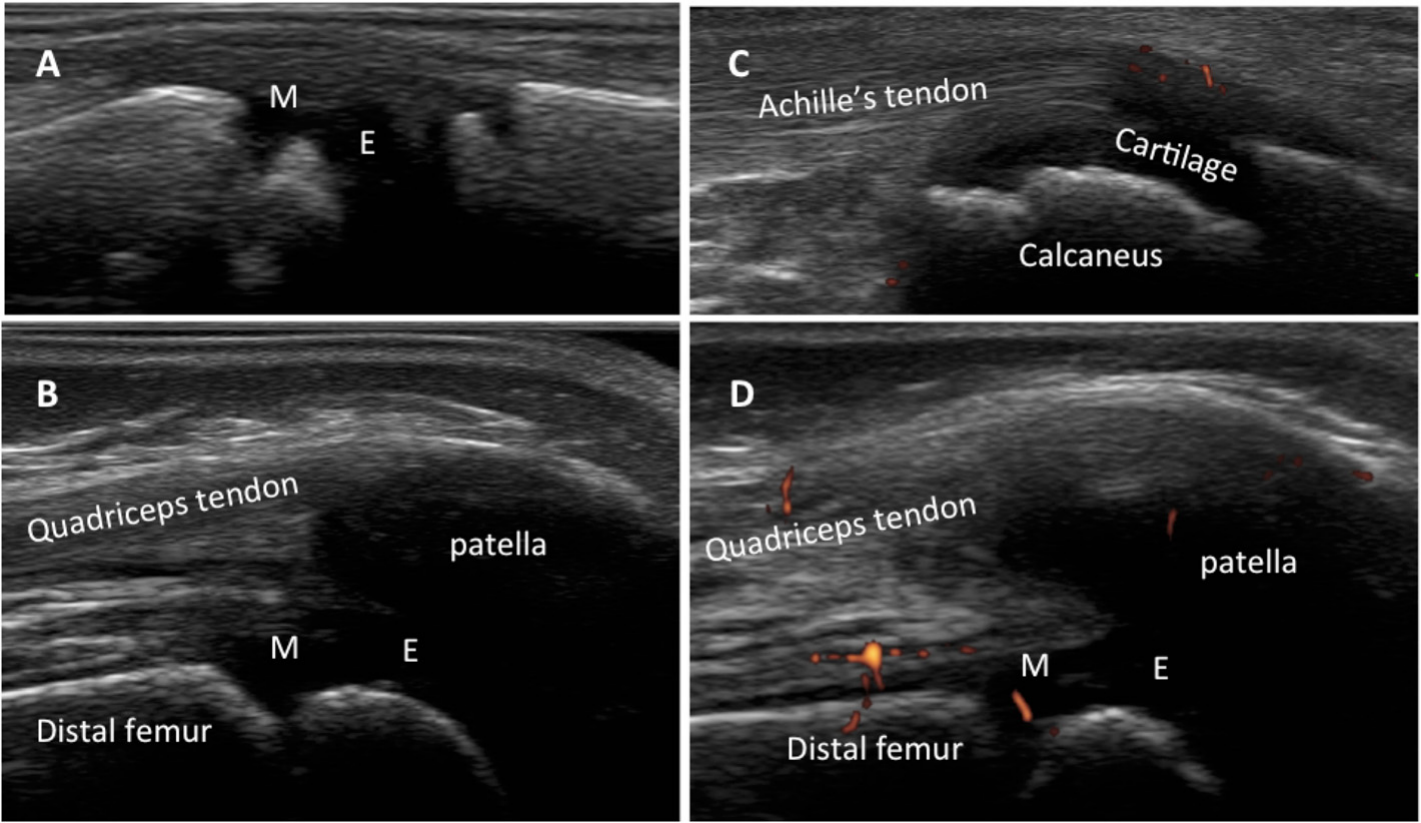
Metaphysis (M) look like erosions; epiphysis (E) and unossified bones are anechoic, like synovial effusion (**a**,**b**). Physiological vascularization at insertion of enthesis to the cartilage can be frequently detected, resembling enthesitis (**c**). Feeding vessels can be intraarticular or close to ossifying nuclei, and must not be considered as signs of active synovitis (**d**). **a**. Dorsal longitudinal scan of the II metacarpophalangeal joint in a 2 years old child, *grey-scale*. **b**. Longitudinal medial scan of the suprapatellar recess in a 2 years old child, *grey-scale*. **c**. Longitudinal scan of the Achilles tendon at insertion to the calcaneus in a 5 years old child, *power Doppler* **d**. Longitudinal medial scan of the suprapatellar recess in a 5 years old child, *power Doppler*

When dealing with musculoskeletal imaging in childhood, it is noteworthy to emphasize the unique feature of the growing skeleton, which include age-related variation of the thickness of the articular cartilage and incomplete ossification. Moreover, in children the epiphysis are vascularized and metaphyseal vessels anastomose with epiphyseal vessels through the growth plate. Depending on the properties and sensitivity of the ultrasound machine, vascularization in this area can be physiologically detected by MSUS in healthy children [12], whereas it would be regarded as pathological in adults. Several pitfalls can lead the ultrasonographer with little experience in pediatric joints to embarrassing misinterpretation of images (Fig. 3). Therefore, the awareness of a high ratio cartilage/bone, that changes during child growth, and the anatomical knowledge of the feeding vessels are of foremost value and cannot be overtaken.

### Usefulness of MSUS in children

Despite several challenges, MSUS is commonly regarded as a very useful tool in children, as outlined by the results of recent national and international surveys among pediatric rheumatologists [13, 14]. For most of the respondents, MSUS owned particular relevance for the ability to detect subclinical synovitis and to improve the classification of patients in JIA subtypes, as guidance to intraarticular corticosteroid injections and for capturing early articular damage. In addition, some specific joints were considered as most suited to be studied by MSUS, specifically the midfoot, the ankle, the hip, the wrist, the small joints of hands and feet.

### What is the evidence for usefulness of MSUS in JIA?

#### Subclinical synovitis and tenosynovitis

**Fig. 4:**
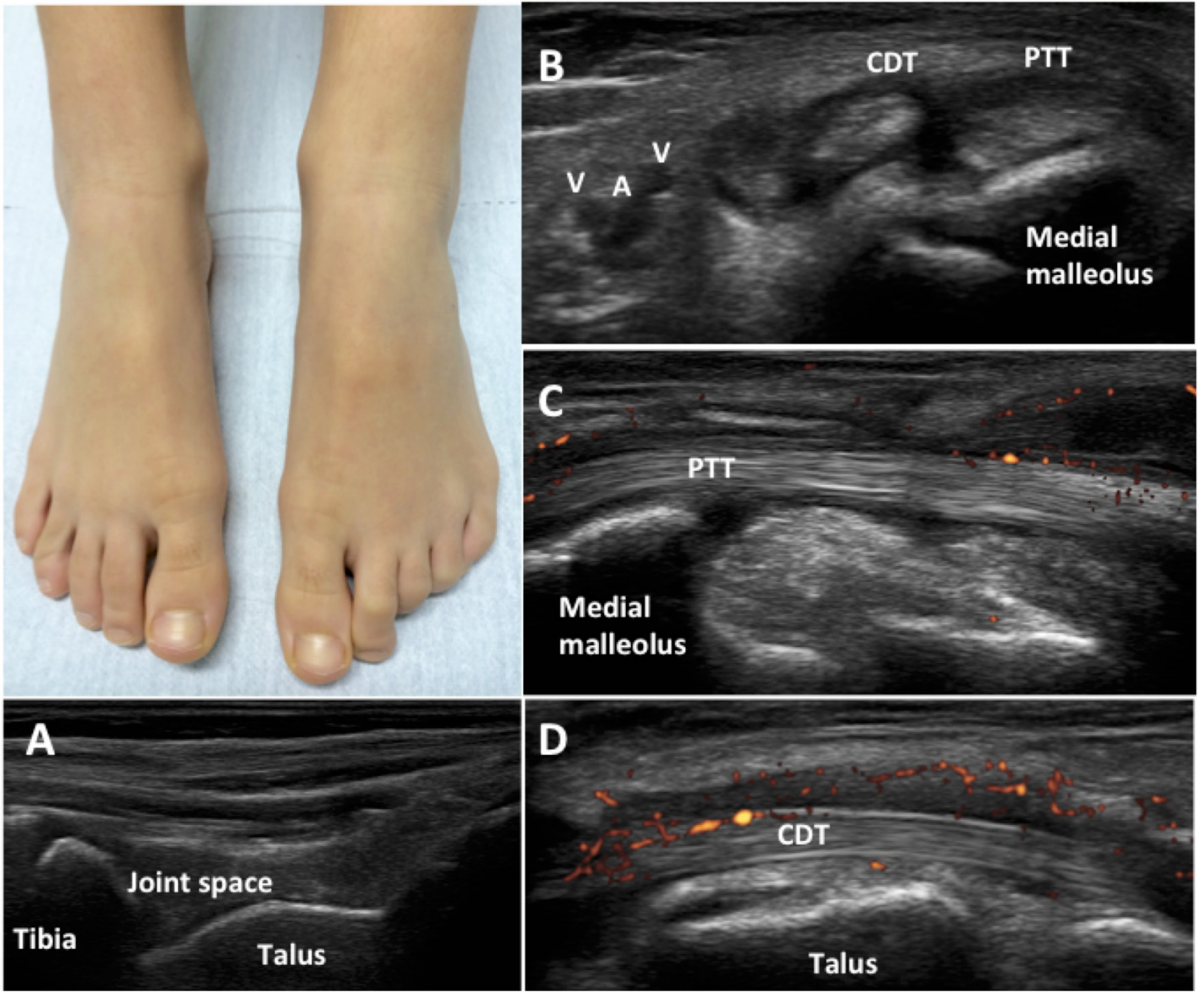
A 10 years old girl with JIA presented with mild swelling of the left ankle, without tenderness/pain on motion or limitation on motion. MSUS showed no signs of synovitis at the tibiotalar joint (**a**), and allowed detection of a hypo-anechoic halo around the medial tendons (**b**) and pathologic vascularization on power Doppler along both the posterior tibialis tendon (**c**) and the common flexor digiti tendon (**d**), indicating flourishing tenosynovitis. PTT: posterior tibialis tendon; CDT: common flexor digitorum tendon; A: posterior tibialis artery; V: posterior tibialis vein

Arthritis in JIA is so far defined as swelling within a joint, or limitation in the range of joint movement with joint pain or tenderness, persistent over time, observed by a physician, and not due to primarily mechanical disorders or to other identifiable causes [15]. Currently, the definition of an oligoarticular or polyarticular involvement is based on the number of active joints. Therefore, complete joint assessment is mandatory for accurate assessment of the disease. However, in the recent years several authors documented a discrepancy between clinical and MSUS examination in detecting synovitis in JIA. In our experience, 1664 joints in 32 children with JIA were evaluated both clinically and with MSUS. A total of 104 (6.3 %) and 167 (10 %) joints had clinical and MSUS synovitis, respectively. Of the 1560 clinically normal joints, 86 (5.5 %) had synovitis on MSUS. The frequency of subclinical synovitis was greater in wrists, PIP, subtalar and foot joints [16]. Haslam et al. compared clinical and MSUS evaluation in 680 joints of 17 patients with early (<12 months) oligoarticular JIA. Six children had subclinical synovitis, more frequently detected in the small joints of hands and feet [17]. Other authors reported similar findings in the assessment of peripheral joints and the ankle [18–20]. In particular, Rooney at al. observed that the clinical examination might not be able to distinguish whether joint swelling in the ankle is due to synovitis, tenosynovitis or both (Fig. 4). In 34 JIA patients who had clinically detected swelling in 49 ankles they evaluated the prevalence of MSUS synovitis and tenosynovitis. Only 29 % of ankles had tibiotalar effusion alone, whereas tenosynovitis associated with tibiotalar synovitis and tenosynovitis alone were detected in 71 and 39 % of ankles, respectively. Concomitant tenosynovitis and tibiotalar effusion were found in 33 % of ankles [20]. In another study of the same group, 32 % of the ankles considered clinically involved did not show MSUS synovitis. In 42 % of ankles recorded as clinically normal MSUS detected involvement of medial tendons, whereas less than 50 % of the lateral tendons deemed to be clinically involved were affected on MSUS [21].

These findings highlight that clinical examination in JIA may underestimate the extension of synovitis and sometimes cannot be precise, whereas MSUS can improve the sensitivity and the accuracy in the detection of the exact sites of inflammation in the joint. Therefore, implementation of clinical examination with MSUS in children with JIA can lead to important implications in therapeutic decisions (i.e. indication to a second line drug or biologic treatment, or exact location of intraarticular corticosteroid injections) and for monitoring treatment efficacy.

#### Enthesitis

Enthesitis represents the main feature of the enthesitis-related arthritis (ERA) JIA subgroup, according to the International League of Associations for Rheumatology (ILAR) classification of JIA, and is clinically defined as tenderness at the insertion of a tendon, ligament, joint capsule, or fascia to bone [15]. The clinical demonstration of enthesitis in children is challenging owing to the peculiar fat distribution, that can mask the anatomical landmarks, and the frequently insufficient cooperation of very young children. A recent study reported on MSUS sensitivity to detect enthesitis in children with JIA [22]. The authors compared physical examination and power Doppler (PD) MSUS in detecting enthesitis in five sites (the quadriceps tendon insertion, the proximal and the distal patellar ligament insertion on the tibial tuberosity, the Achilles tendon insertion on the posterior surface of the calcaneus, and the plantar fascia insertion) in 26 patients with JIA and 41 healthy children. None of the healthy children had PD MSUS evidence of enthesitis. In patients with JIA physical examination showed enthesitis at only 12.5 % of sites, whereas PD MSUS enthesitis was found at 9.4 % of the investigated sites. Clinical enthesitis was often associated with PD MSUS enthesitis. On the other hand, 50 % of the sites exhibiting PD MSUS were clinically normal. Of note, 20 % of the sites with PD MSUS enthesitis were in patients with oligoarticular JIA and 10 % in patients with polyarticular JIA.

Recent studies confirmed a higher sensitivity of MSUS in comparison with clinical examination in detecting enthesitis in different entheseal sites in patients with ERA-JIA [23, 24].

**Fig. 5:**
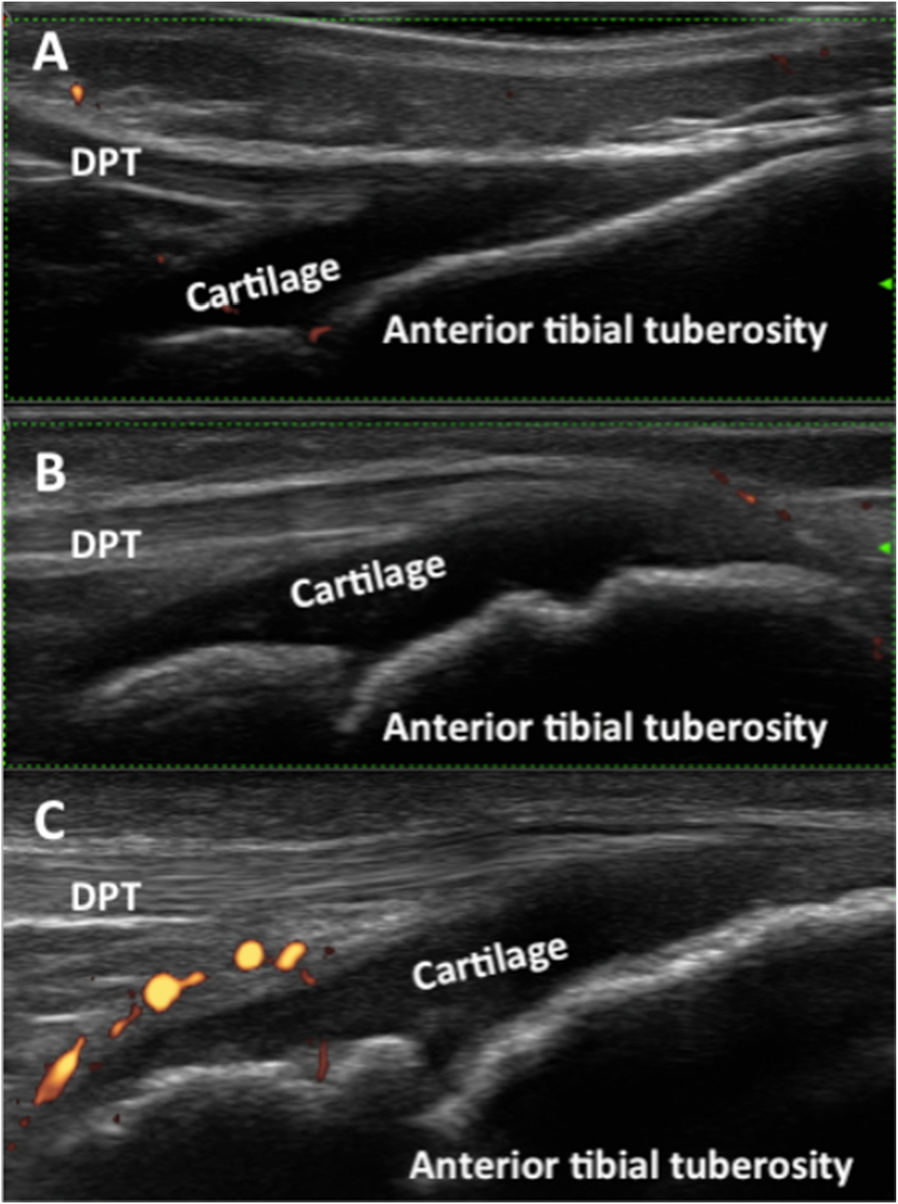
Sonographic appearance of the patellar enthesis at different ages. **a**. 2 years old girl; **b**. 8 years old girl; **c**. 10 years old boy. DPT: distal patellar tendon

These findings overall indicate that MSUS may help to detect clinically silent peripheral enthesitis in ERA and non-ERA patients, and can contribute substantially to the correct diagnosis and classification of JIA. On the other hand, comprehensive knowledge of the ultrasonographic appearance of entheses in healthy subjects during the developmental ages is still under investigation (Fig. 5) and reliable definitions for normal and pathological MSUS findings in pediatric entheses need first to be addressed.

#### ILAR classification JIA subgroups

The most recent ILAR classification criteria for JIA were developed to delineate homogeneous subtypes of JIA for research purposes [15] and so far have been adopted to stratify patients and select candidates to second-line treatment or biologic therapy [25–31]. In particular, children with JIA are classified as having oligoarthritis or polyarthritis based on the number of affected joints; the presence of active arthritis in at least five joints is a requisite for the definition of a polyarticular disease course and for the eligibility to second-line or biologic agents in several health systems. As already mentioned, MSUS demonstrates higher sensitivity in detecting synovitis than the clinical examination, and, as a consequence, can lead to reclassify patients, with a trend towards a more extensive joint involvement and potential more aggressive therapy. In the author’s experience, of 32 children with JIA evaluated cross-sectionally both with clinical and MSUS examinations, five patients, classified as having mono/oligoarthritis involvement by clinical examination, showed a polyarthritis involvement based on MSUS findings [16]. Similarly, one patient out of 17 children with oligoarticular JIA was reclassified as having polyarthritis after MSUS examination by Haslam et al. [17]. Of note, MSUS could precisely identify inflammatory tendon involvement in JIA patients with ankle arthritis, as outlined by Rooney at al [20, 21]. Though tendons can be affected throughout the whole course of JIA, the current ILAR classification does not take into consideration tendon involvement. Further, MSUS enthesitis could be found in both in ERA and non-ERA-JIA patients, as mentioned above [22–24].

Overall these findings suggest that the use of MSUS may yield important insights in the location of inflammatory changes in joints and in different JIA subtypes, providing the anatomic rationale for a future refinement of the classification of childhood arthritis.

#### Definition of disease remission

It has been recently argued that remission of JIA defined on clinical grounds does not couple with remission defined with imaging [32–34]. However, the clinical significance and prognostic value of this finding is unclear, as the presence of MSUS abnormalities, including PD signal, in patients with clinically defined inactive disease did not predict subsequent synovitis flare [35]. This finding contrasts with observations in adults with rheumatoid arthritis (RA), in which vascularization detected by PD MSUS predicted short­term disease flare after clinical remission [36, 37].

These puzzling MSUS features may be due to underlying immunopathological mechanism and local changes peculiar of JIA, still unknown and different to the classical autoinflammatory process in seropositive RA [38]. Future research oriented to explore this supposition and correlation of MSUS with biologic markers of disease activity [39, 40] is advisable.

#### Imaging guided injections

**Fig. 6:**
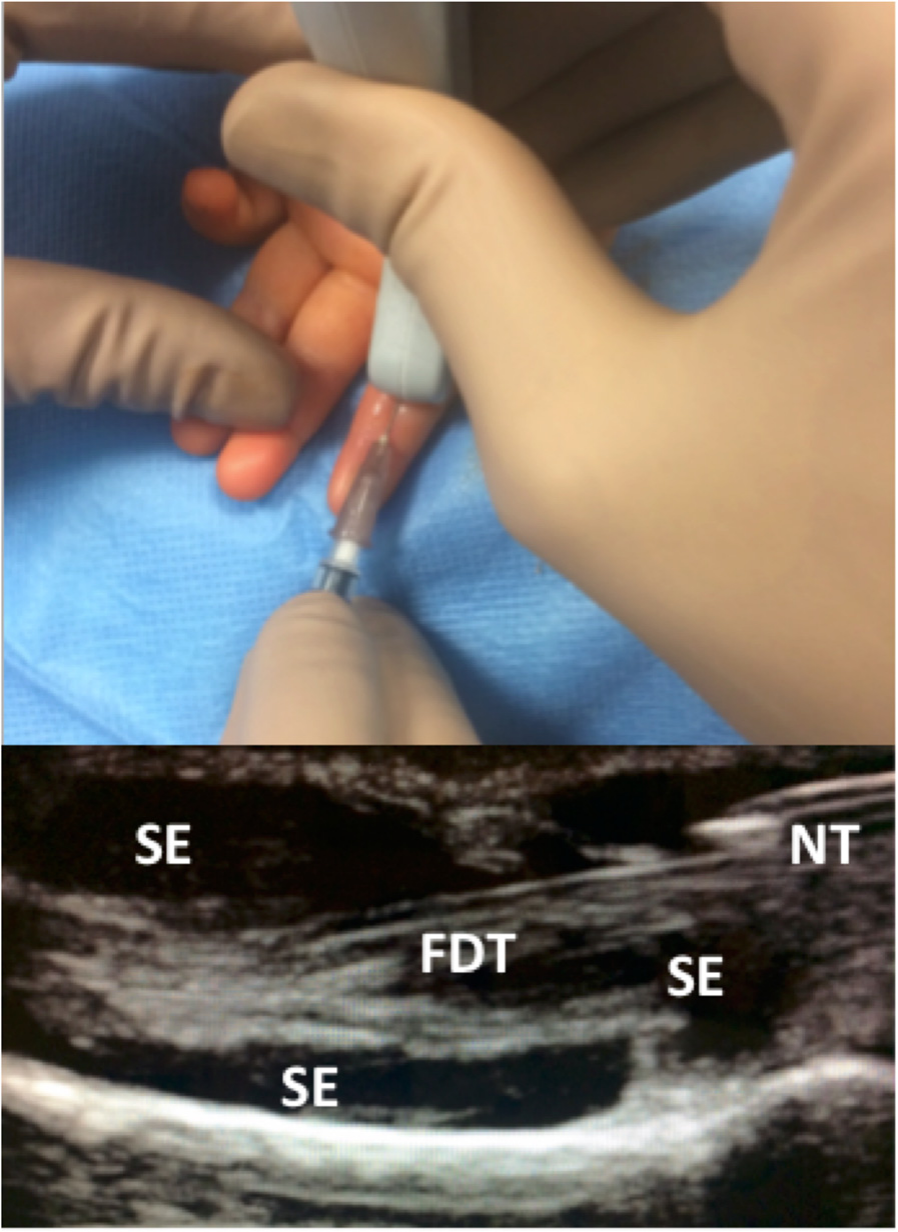
MSUS allows detection of the exact location of inflammation and correct needle placement, even in very small sites, such as the synovial sheath of the flexor tendon of the VI digit in a 3 years old girl. NT: needle tip; FDT: flexor digiti tendon; SE: synovial effusion

Intra-articular corticosteroid injections (IACI) are widely used in JIA to induce prompt relief of symptoms of active synovitis. Blind-method is sometimes difficult in the younger, due to the small joint size and the subcutaneous fat masking bony landmarks. Therefore MSUS may represent a fundamental tool, not only for the precise detection of the inflamed area, but also for the accurate placement of the needle tip within the different affected anatomical structures, in order to maximize the treatment efficacy and minimize local side effects (mainly subcutaneous atrophy or local skin hypopigmentation) [41] (Fig. 6). Several authors documented the correct needle placement and the efficacy of MSUS-guided injections of clinically difficult to access joints, such as the hip [42], or clinically difficult to assess joints, such as the ankle and the midfoot [43]. In particular, Laurell at al. performed MSUS-guided IACI in 85 compartments of the ankle, including twenty-one tendon sheaths and a ganglion cyst, and observed normalization or regression of MSUS-detected synovial hypertrophy in 89 % of the injected sites at 4 weeks after IACI. The same authors also reported on the efficacy of MSUS-guided IACI in 21 compartments of 15 wrists, with increasing improvement of synovial hypertrophy and synovial hyperemia at 1 week and 4 weeks post-injection [44]. Another joint that can particularly benefit from MSUS-guided IACI is the temporomadibular joint (TMJ). Efficacy and safety assessed 6–8 weeks post MSUS-guided injection of 63 TMJ in 39 children with JIA showed improvement in all symptoms and only one side effect (local scar) in one patient [45]. However, data on correct needle placement in TMJ by MSUS-guidance are scarce and controversial [46, 47], and need to be further investigated.

#### Cartilage damage

Joint cartilage is a known target in inflammatory arthritis. Though MSUS do not allow to visualizing cartilage in its entireness in all joints, due to its limited acoustic window, loss of MSUS detected cartilage in “key sites” of the joints may represent an early marker of damage in JIA. Spannow, et al were the first to attempt the measurement and quantification of cartilage thickness in pediatric subjects [48]. They provided normal ranges of MSUS-detected cartilage thickness in small and large joints of healthy children, and designed a complicated formula in order to calculate cartilage thickness in the clinically dominant joints for different age groups of children [49]. Cartilage was found significantly thicker in boys than in girls and diminished progressively with age in both sexes, as similarly reported later by other authors [50]. The same investigators provided data on intra- and interobserver agreement and validated MSUS measurement of cartilage thickness in several joints of healthy children by comparing MSUS with findings obtained with magnetic resonance imaging (MRI), with an overall good agreement except for the wrist [51, 52]. In a subsequent study, Spannow and coworkers measured cartilage thickness with MSUS in joints of patients with JIA, and compared the findings with those obtained in healthy children. Of note, cartilage thickness in joints of patients with JIA was significantly lower than in the healthy cohort, regardless of whether the examined joints have been previously affected by arthritis [53]. Pradsgaard, et al. from the same group of investigators, compared MSUS and MRI measurements of distal femoral cartilage thickness in children with JIA. They identified the intercondylar notch as the best site to assess cartilage thickness, because of its easier assessment and lower variability on MSUS as compared to MRI [54]. These findings may support that irreversible changes can occur in cartilage of children with JIA despite the localization of synovial inflammation. The measurement of cartilage thickness at the intercondylar notch of the knee may act as a surrogate of overall early cartilage damage in JIA patients. However, since MSUS and MRI cartilage measurements at different sites of the knee were problematic in the previously affected and unaffected knees compared to the whole cohort, this proposal is controversial [55]. Thorough studies in patients with long term inactive and active JIA and comparison with the newer MRI techniques for the biochemical evaluation of cartilage matrix composition over time [56] would give insights to address these challenging issues.

#### Bone erosions

**Fig. 7:**
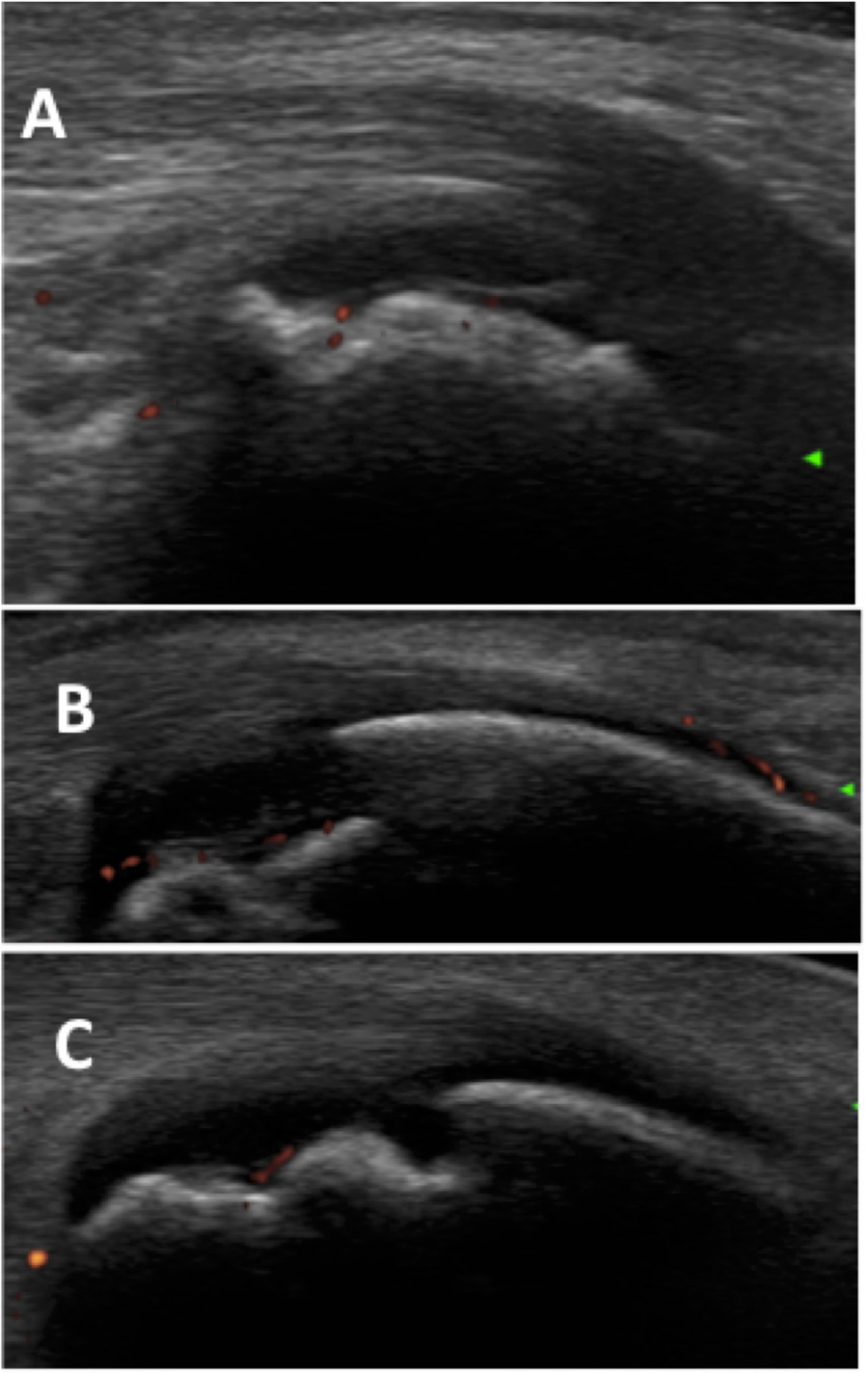
The calcaneus bone appears fragmented in children respectively aged 6 years (**a**), 8 years (**b**), and 10 years (**c**). Feeding vessels can be detected by power Doppler (*red* dots)

Compared with conventional radiography, the capacity to assess dynamically and in real-time the joints in several planes makes MSUS a more useful tool for detecting erosions [57, 58]. The OMERACT (Outcome Measures in Rheumatology Clinical Trials) definitions of bone erosion require documenting an interruption of the intraarticular bone surface or cortical breaks with a step-off bone defect visible in at least two perpendicular planes [59]. However, children anatomy is characterized by physiological bone irregularities due to the presence of ossification centers and growth plates at the epiphyseal cartilage. Moreover, when ossified, some bones can at first appear fragmented and irregular (Fig. 7). Consequently, in children all these peculiar normal findings may potentially be misinterpreted as cortical erosions by inexperienced operator eyes. Further, due to the peculiar vascularization of the epiphysis in children, that anastomoses with metaphyseal vessels through the growth plate, an inflammation affecting the epiphyseal cartilage may spread to the ossification center, causing excessive growth, deformities, or destruction with epiphyseal erosions rather than marginal erosions, unlike RA patients. These observations advise that further validation and large-scale studies are required to determine the potential role and the accuracy of MSUS in the detection of bone damage in children.

### Advances and perspectives

At present, increasing interest in the use of MSUS during daily clinical practice is spreading among pediatric rheumatologists. Nonetheless, several topics are still under investigation or need to be further addressed. First of all, the anatomical specificities of the growing skeleton in children make imaging interpretation more challenging than in adults and thorough knowledge of the sonoanatomy changes at the different growth ages is essential to distinguish physiological appearance from pathological findings. Recently, definitions for gray scale sonographic features of joints in healthy children have been proposed and validated [60]. Sonographic appearance of tendons, entheses and Doppler MSUS in healthy children was not described and requires specific additional studies. Secondly, in the recent years several investigators proposed to assess children using standard scans according to the EULAR (European League Against Rheumatism) guidelines [61]. However, this scanning approach has been developed for MSUS examination of adults, and its applicability and reliability in children were not demonstrated. In the frame of the OMERACT Ultrasound pediatric subtask force, Collado et al. set up a standardized MSUS examination method specific for the pediatric population [12]. The study showed a high quality of images obtained by all the investigators, despite the use of different sonographic equipments, indicating suited methodology. MSUS standard scans proposed were appropriate and reproducible in children regardless of their age. Further, the standardized MSUS examination, together with the MSUS definitions of normal features in pediatric joints, enabled the investigators to achieve information on physiological blood flow and on age-related changes of bones in each joint. The next step would be standardization of findings in synovitis and in abnormal vascularization detected by Doppler, and is already ongoing.

Studies in adults with RA have recommended the use of reduced and simplified joint counts for MSUS assessment of disease activity, preventing in this way too long-lasting evaluations [62, 63]. This may be particularly relevant in children, since they notoriously are less tolerant than adults of undergoing investigations. Recently, some investigators proposed a reduced joint PD MSUS assessment and provided preliminary evidence of its validity, reliability, sensitivity to change and feasibility in evaluating synovitis in JIA [64]. Nonetheless, these results should be confirmed in independent cohorts with large number of patients. Additional studies in children with JIA in order to define the pattern and number of joints to be assessed with MSUS, both with grey scale and Doppler, are warranted.

Although there is an astounding interest in the application of MSUS in children, only few pediatric rheumatologists are able to perform MSUS on their own, as outlined by recent surveys [13, 14]. On the other hand, most of the respondents to the surveys stated their willingness to embrace this imaging modality and to gain experience in performing MSUS by themselves. Therefore, access to suitable training and mentorship for MSUS in children is of primary relevance. Further educational activities are required to enhance the use of this imaging modality in pediatric rheumatologists’ practice.

## Conclusions

MSUS shows great promise in the assessment and management of children with JIA, so that it is likely to play an increasing role in clinical practice of pediatric rheumatologists, as in adult rheumatology. However, several issues, including the anatomical knowledge of sonographic changes over time and the underlying immunopathophysiology, the standardization and validation of MSUS in healthy children and in patients with JIA, are still under investigation. Currently, few pediatric rheumatologists are able to perform MSUS on their own. In the future further research and educational efforts are required for expanding this imaging modality to more clinicians in their daily practice.

## Key points

- MSUS can detect inflammatory changes more frequently than the clinical examination and can visualize their exact location in joints of children with different JIA ILAR subtypes, yielding important insights in the extent, pathophysiology and classification of childhood arthritis.
- Due to the unique features of the growing skeleton, accurate knowledge of MSUS findings over time in children is warranted. Currently, validated definitions of MSUS features in joints of healthy children are available; standardization of scanning techniques specific for the pediatric population and definitions of MSUS pathological findings in children are ongoing.
- Correlation of MSUS findings with biologic markers, clinical features and other imaging tools should be studied prospectively to investigate the clinical meaning and prognostic value of MSUS-detected abnormalities in different joints at different time points of the disease course.
- Despite the astounding interest in the use of MSUS in children, only few pediatric rheumatologists currently perform MSUS on their own. Further educational activities for suitable training and mentorship in MSUS in children are advisable in the future.

## Abbreviations

MSUS, musculoskeletal ultrasound; JIA, juvenile idiopathic arthritis; ERA, enthesitis-related-arthritis; PD, power Doppler; ILAR, International League of Associations for Rheumatology; RA, rheumatoid arthritis; IACI, intraarticular corticosteroid injection(s); TMJ, temporo-mandibular joint(s); MRI, magnetic resonance imaging; OMERACT, Outcome Measures in Rheumatology Clinical Trials; EULAR, European League Against Rheumatism.
